# Treatment of Fracture-Related Infections with Bone Abscess Formation after K-Wire Fixation of Pediatric Distal Radius Fractures in Adolescents—A Report of Two Clinical Cases

**DOI:** 10.3390/children10030581

**Published:** 2023-03-18

**Authors:** Markus Scharf, Nike Walter, Markus Rupp, Volker Alt

**Affiliations:** 1 Department for Trauma Surgery, University Hospital Regensburg, 93053 Regensburg, Germany; 2 Department for Psychosomatic Medicine, University Hospital Regensburg, 93053 Regensburg, Germany

**Keywords:** fracture-related infection, FRI, radius, K-wire, spacer

## Abstract

Closed reduction and K-wire fixation of displaced distal radius fractures in children and adolescents is an established and successful surgical procedure. Fracture-related infections after K-wire fixation are rare but can have significant consequences for the patient. There is a lack of literature on the treatment of K-wire-associated fracture-related infections in children and adolescents. Herein, we report two cases of fracture-related infection after initial closed reduction and Kirschner wire fixation in two adolescents. One 13-year-old boy and one 11-year-old girl were seen for fracture-related infections 4 and 8 weeks after closed reduction and percutaneous K-wire fixation of a distal radius, respectively. X-ray and magnetic resonance imaging (MRI) revealed a healed fracture with osteolytic changes in the metaphyseal radius with periosteal reaction and abscess formation of the surrounding soft tissue structures. A two-staged procedure was performed with adequate debridement of the bone and dead space management with an antibiotic-loaded polymethyl methacrylate (PMMA) spacer at stage 1. After infection control, the spacer was removed and the defect was filled with autologous bone in one case and with a calcium sulphate–hydroxyapatite biomaterial in the other case. In each of the two patients, the infection was controlled and a stable consolidation of the distal radius in good alignment was achieved. In one case, the epiphyseal plate was impaired by the infection and premature closure of the epiphyseal plate was noted resulting in a post-infection ulna plus variant. In conclusion, a fracture-related infection after Kirschner wire fixation of pediatric distal radius fractures is a rare complication but can occur. A two-stage procedure with infection control and subsequent bone defect reconstruction was successful in the presented two cases. Premature closure of the epiphyseal growth plate of the distal radius is a potential complication.

## 1. Introduction

Fractures of the forearm are regularly seen in children, accounting for 74% of paediatric fractures of the upper extremity [1]. For distal forearm fractures, an incidence of 738/100,000 persons/year has been reported, whereby around 2% of males aged 13 years sustain a forearm fracture each year [2]. While most fractures are managed conservatively, surgical treatment is recommend in some cases, where adequate reduction or adequate stabilization of the fractures cannot be achieved by closed reduction alone [3]. In these cases, the standard surgical treatment is closed reduction and fracture fixation by percutaneous K-wire pinning with a good treatment outcome [4].

All surgical procedures for fracture treatment bear the risk of a fracture-related infection (FRI). The term FRI was introduced and adopted in the last year for all types of infection complication in fracture care treatment [5].

Even though this complication remains rare with a rate of 1–2% for closed fractures in adults [6], the management is challenging and often requires a multidisciplinary approach [7]. Key for treatment success is an adequate surgical debridement with accompanying local and systemic antibiotic therapy for infection control and subsequent bone healing [8].

While superficial K-wire fixation after closed reduction and percutaneous placement of the K-wires can frequently lead to superficial soft tissue infections in children, which can easily be mastered by removal of the affected K-wire, bone infections with intraosseous abscess formation in these cases remains extremely rare.

To the authors’ best knowledge, no literature is available on the treatment of FRI with bone sequester formation at the distal radius after percutaneous pinning of a distal radius fracture in children. The purpose of this article is to present two clinical cases of FRI with intraosseous abscess formation at the distal radius after percutaneous pinning of distal radius fractures in adolescents including a two-staged treatment protocol with surgical debridement for infection control at stage one and subsequent bone reconstruction at stage two.

## 2. Case Presentations

### 2.1. Case 1

**Figure 1:**
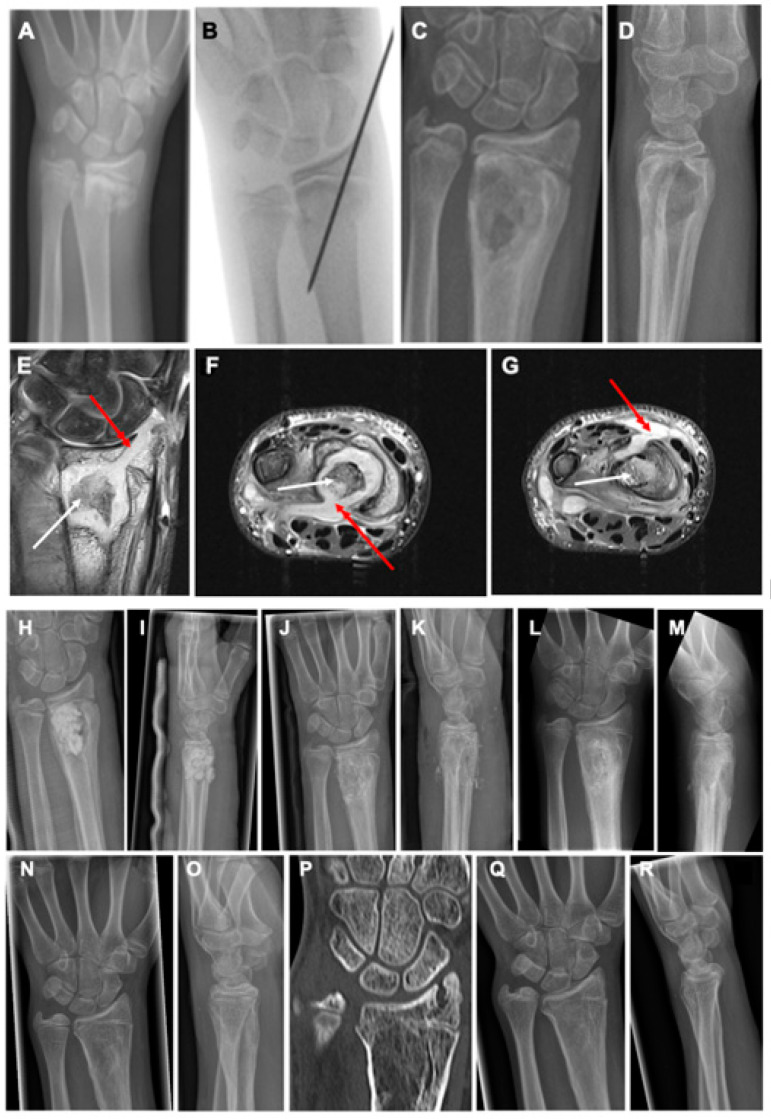
A 13-year-old male patient sustained a dislocated fracture of the distal radius and an undislocated distal ulna fracture after a bicycle accident (**A**). Closed reduction and percutaneous K-wire fixation was performed with epicutaneous placement of the K-wire (**B**). Four weeks after surgery, the boy presented with acute redness and swelling at the distal forearm with high suspicion of a fracture-related infection. The K-wire was completely loosened and removed before X-ray control (**C**,**D**). X-rays showed a healed fracture with severe osteolysis in the metaphyseal region with accompanying periosteal reaction. MRI confirmed severe osteolysis in the metaphyseal region with more details, such as abscess and sequestrum formation (white arrows) and affection of the growth plate and epiphysis through the channel, where the K-wire had initially been placed on coronal views ((**E**), red arrow). Although the initial K-wire placement had violated the radiocarpal joint, no signs of septic arthritis of the wrist joint were detected. Axial MRI images showed cortical lysis at the metaphyseal area with abscess penetration into palmar ((**F**), red arrow) and dorsal ((**G**), red arrow) soft tissue structures. Treatment consisted of a two-stage procedure with debridement and sequestrectomy, partial synovectomy, and insertion of an antibiotic-loaded PMMA spacer at stage 1 (**H**,**I**). After 4 weeks, the spacer was removed and the defect was filled with autogenous bone graft from the ipsilateral iliac crest (**J**,**K**). After six weeks, the bone grafting procedure showed good new bone formation in the former infected defect area without recurrence of infection but with first signs of premature closure of the ulnar aspect of the epiphyseal growth plate (**L**,**M**). After a total of six months after bone grafting, the metaphyseal bone showed complete healing with remodeling of the cortical area and disappearance of the initial periosteal reaction with progressive closure of growth plate with correct alignment of the distal radioulnar joint (**N**,**O**). CT scan on the same day confirmed the successful bone regeneration in the metaphyseal zone with remodeling of the cortical area complete and premature closure of approximately 80% of the epiphyseal plate with still correct appearance of the distal radioulnar joint (**P**). At the 18 months follow-up, premature arrest of the epiphyseal plate resulted in an ulna plus variant of the wrist joint (**Q**,**R**).

A 13-year-old boy sustained a distal forearm fracture with a dislocated fracture of the distal radius in combination with an undislocated distal ulna fracture after a bicycle accident (Figure 1). Closed reduction and percutaneous K-wire of the distal radius fracture was performed in an external hospital. Perioperative infection prophylaxis with 2 g cefazoline was performed and a K-wire was placed epicutaneously. Four weeks after surgery, the boy presented to our emergency department with acute redness and swelling at the distal forearm with purulent drainage from the K-wire skin incision. The loosened K-wire was removed prior to X-ray control. X-rays showed a healed fracture at the distal radius with osteolysis in the central metaphyseal region with accompanying periosteal reaction. Magnetic resonance imaging (MRI) confirmed severe osteolysis in the metaphyseal region with abscess and sequestrum formation in the central metaphyseal region in combination with cortical lysis and abscess penetration into palmar and dorsal soft tissue structures. MRI also showed affection of the growth plate and epiphysis through the channel, where the K-wire had initially been placed. Although the initial K-wire placement had violated the radiocarpal joint, no signs of septic arthritis of the wrist joint were detected.

Treatment consisted of a two-stage procedure with debridement and sequestrectomy, and partial synovectomy, and the insertion of an antibiotic-loaded polymethyl methacrylate (PMMA) spacer (mixture: 40 g Copal^®^, Heraeus, Wehrheim, Germany + 2 g vancomycin powder) at stage 1 via a modified Henry approach was performed. Approximately, 7.5 cc of the antibiotic-loaded PMMA was used. In addition, a 2 cm lateral approach to the processus styloideus radius was needed for full soft tissue and bone debridement. Deep tissue samples taken intraoperatively from the deep soft tissue layer and the bone revealed culture growth of *Staphylococcus aureus*. After 4 weeks, the spacer was removed and the defect was filled with autogenous bone graft from the ipsilateral iliac crest. The patient received cefazolin (100 mg/kgKG) three times daily i.v. for a duration of seven days according to the resistogram of the pathogen. Sport activities were restricted for three months.

The patient remained infection free, and after six weeks, the bone grafting procedure showed good new bone formation in the former infected defect area without recurrence of infection but with the first signs of premature closure of the ulnar aspect of the epiphyseal growth plate.

After a total of six months after bone grafting, X-ray control showed the metaphyseal bone with remodeling of the cortical area and disappearance of the initial periosteal reaction. However, progressive closure of the growth plate had also to be noted. At the 18 months follow-up, premature growth arrest of the epiphyseal plate resulted in an ulna plus variant of the wrist joint. The patient had a range of motion of extension/flexion of 45-0-70° with full pro- and supination.

### 2.2. Case 2

**Figure 2:**
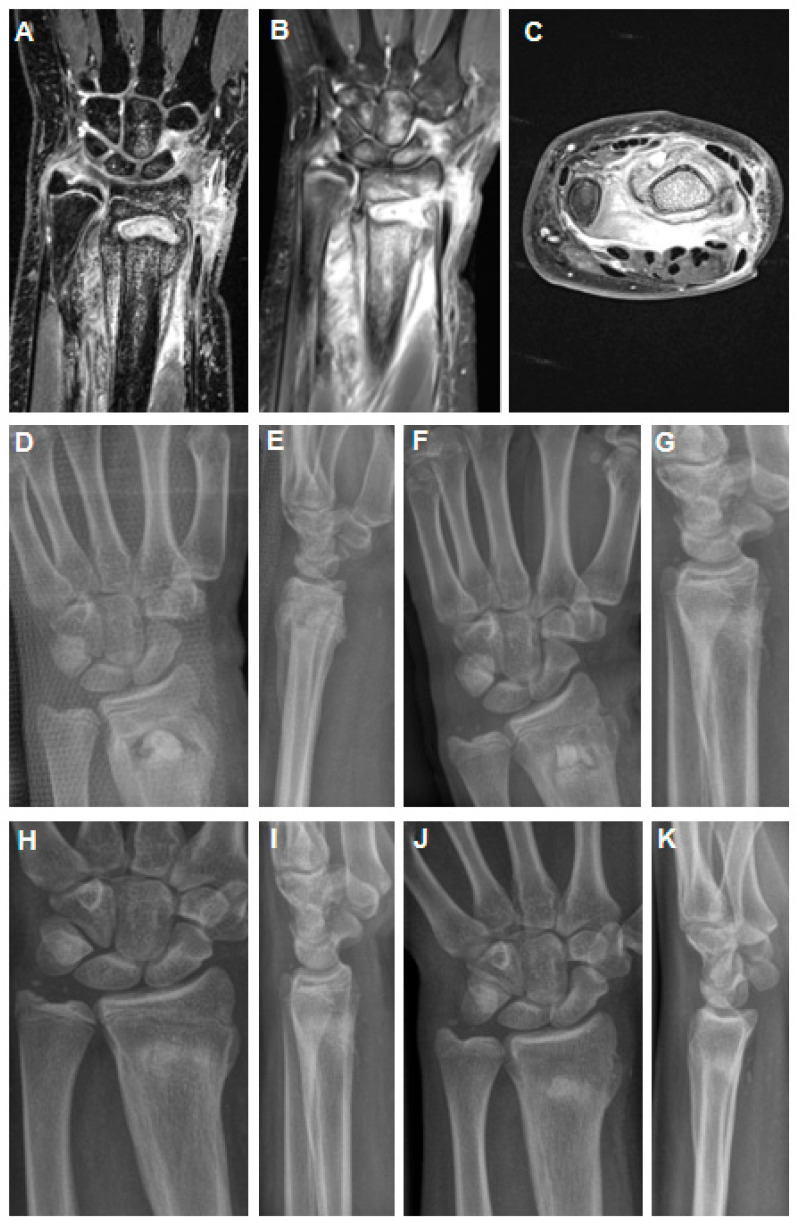
MRI imaging 4 weeks after K-wire removal after initial closed reduction and K-wire fixation of a dislocated distal radius fracture in an 11-year-old girl (**A**–**C**). Abscess formation in the distal metaphysis of the radius with intensive periosteal reaction (**A**,**B**) and affection of the growth through the K-wire channel. Axial section revealed strong abscess into the palmar soft tissues (**C**). Post-operative X-rays show filling of the metaphyseal defect area with a PMMA spacer (**D**,**E**). The spacer was exchanged against a hydroxyapatite–calcium sulphate biomaterial 9 weeks after stage 1 (**F**,**G**). After 6 weeks, there was already good remodeling of the metaphyseal bone zone (**H**,**I**). At midterm follow-up of 16 months, there was correct alignment of the wrist joint with premature closure of the growth plate (**J**,**K**).

An 11-year-old girl was treated in an external hospital by closed reduction and percutaneous K-wire pinning of a displaced distal radius fracture (Figure 2). The two K-wires were placed epicutaneously and were removed after 4 weeks.

She was seen 5 weeks after K-wire removal in our emergency department for a swollen and redness wound at the distal radius. MRI imaging revealed consolidation of the fracture with good alignment of the distal radius and clear abscess formation in the distal radius with extensive periosteal and palmar soft tissue involvement. Affection of the epiphyseal plate by the former K-wire channel was also suggested.

Similarly to case 1, a two-stage protocol was applied with debridement and removal of the abscess from the metaphyseal distal radius and the surrounding soft tissue. A palmar modified Henry approach as well as a dorsal incision were performed to evacuate the abscess formation. A PMMA spacer loaded with gentamicin, clindamycin, and vancomycin was placed (mixture: 40 g Copal^®^, Heraeus, Wehrheim, Germany + 2 g vancomycin powder). Approximately 3 cc of the antibiotic-loaded PMMA was used.

Nine weeks later, stage 2 was performed with removal of the PMMA spacer and the bone defect was filled with three pellets of a hydroxyapatite–calcium sulphate biomaterial loaded with vancomycin (PerOssal^®^, Osartis GmbH, Dieburg, Germany). Perioperatively, the patient received a single shot of 2 g cefazolin. Pain-adapted full weight-bearing was allowed, but a restriction of sport activity involving any contact was given for eight weeks. There was no recurrence of infection and the X-rays showed complete bone defect healing with incorporation of the biomaterial into the metaphyseal area of the distal radius. There was also premature closure of the epiphyseal plate at the last follow-up after 16 months. Range of motion was extension/flexion 70-0-80° with pro-/supination: 80-0-80°.

## 3. Discussion

To the authors’ best knowledge, there are no cases of fracture-related infections with intraosseous abscess formation after closed reduction and percutaneous K-wire fixation after a distal radius fracture in adolescents in the scientific literature so far, which suggests that the risk of this complication is quite low.

In general, the risk of superficial pin track infections after K-wire fixation of pediatric fractures is low with a reported rate of 1.4% (12 out of 884 cases) and 2% (4 out of 202 cases) [9,10]. Articles on surgical treatment with K-wire fixation of pediatric diaphyseal forearm fractures do not mention the sequela of an infection [11,12,13]. In particular, no reports exist on the appearance of intraosseous abscess formation of the metaphyseal region after percutaneous K-wire placement at the distal radius.

This small series of two cases shows that fracture-related infections after closed reduction and percutaneous K-wire fixation with concomitant bone abscess formation can occur. Causes for the development of an FRI can be multifactorial including an interplay of pathogen-related factors such as virulence factors and host-related factors as well as the severity of the injury and the location [14]. In adults, several risk factors are described comprising, for instance, arterial hypertension, diabetes mellitus type II, and chronic kidney failure [15]. However, these are not common in pediatric FRI.

Interestingly, the clinical presentation of the two cases was different as the second case reported clinical symptoms only 5 weeks after planned removal of the K-wires after uneventful fracture and soft tissue healing without the suspicion of infection by that time. In contrast, the first patient showed clinical symptoms with the indwelling K-wire 4 weeks after initial placement.

Both cases highlight that the principles of the diagnosis and treatment are comparable to FRI in adults [8,16]. Diagnosis should rely on the key publication from the FRI consensus group and FRI is defined either by confirmatory or suggestive criteria [17]. Confirmatory criteria include fistula, purulent drainage, and/or the presence of pus, which was found in both cases and confirmed the diagnosis of FRI.

Regarding treatment, both cases were successfully managed with a two-stage approach with control of the infection during stage 1 by adequate bone and soft tissue debridement, irrigation, and placement of an antibiotic-loaded PMMA spacer. In both cases, there was a large bone abscess with sequestrum in the metaphyseal area of the distal radius that was debrided and removed. Dead space management was performed through an antibiotic-loaded PMMA spacer that additionally allowed local delivery of antibiotics. Good infection control with successful bone reconstruction and subsequent adequate bone remodeling in both cases can also likely be related to the well-vascularized area of the distal radius, particularly in children. This fact might potentially allow for a one-stage approach with debridement and bone void filling with an antibiotic-loaded biomaterial [18].

The removal of indwelling implants due to biofilm formation is another basic step during the initial surgical treatment. However, the K-wires had completely loosened, most likely due to the ongoing infection process, and were removed by simple removal extraction before surgery.

In both cases, *Staphylococcus aureus* was detected, which is in line with the fact that *Staphylococcus aureus* is the most frequently detected strain in FRI in adults [19]. Current recommendations of an initial empiric broad-spectrum therapy before the causing pathogen is identified include a lipopeptide or glycopeptide and an agent-covering Gram-negative bacilli for adults [20]. Other studies have shown that the combination of a glycopeptide such as vancomycin with broad-spectrum antibiotics, such as meropenem, achieved high efficiency in FRI treatment [21]. However, in pediatric FRIs, antibiotic treatment must be adopted to prevent potential side effects. 

Fortunately, the fractures had already consolidated at the time of the clinical appearance of infection signs, and intraoperatively, assessment showed stable bone consolidation. No additional internal or external stabilization of the distal radius had to be performed. This facilitated the further treatment compared to a potential infected non-union situation, in which non-union healing would have had to be achieved additionally.

MRI imaging showed, in both cases, affection of the growth plate of the distal radius that resulted in premature arrest of the growth plate, which is most likely related to the underlying bone infection process at the metaphyseal region with affection of the epiphyseal growth plate. The patient might require shortening of the distal ulna due to a significant ulna plus variant in the future. Interestingly, despite the affection of the epiphyseal plate and epiphysis in both cases, there was no septic arthritis of the radiocarpal or distal radioulnar joint.

## 4. Conclusions

The development of FRI with bone abscess formation at the distal radius after closed reduction and percutaneous K-wiring of pediatric distal radius fractures can occur. They can even present after uneventful fracture healing several weeks after planned K-wire removal. Pre-operative diagnosis is possible by MRI in these cases that is also very helpful to determine the overall bone and soft tissue affection. A two-stage procedure can be deemed successful for infection control and subsequent bone reconstruction. It should be noted that premature closure of the epiphyseal growth plate can occur and close clinical and radiographical follow-up is required in these patients.

## Data Availability

The data that support the findings of this study are available on request from the corresponding author.
